# CT-guided percutaneous aspiration and bleomycin sclerotherapy for symptomatic hepatic cysts: technique and three-year outcomes

**DOI:** 10.3389/fmed.2025.1687375

**Published:** 2025-11-13

**Authors:** Long Li, Yong-Hao Lao, Qiang-Lin Han

**Affiliations:** 1 Division of Interventional Radiology and Endovascular Surgery, Department of Medical Imaging, The Affiliated Guangzhou Twelfth People’s Hospital, Guangzhou Medical University, Guangzhou, Guangdong, China; 2 Division of Interventional Radiology, Department of Medical Imaging, Guangdong Provincial Corps Hospital of Chinese People’s Armed Police Forces, Guangzhou, Guangdong, China

**Keywords:** simple hepatic cyst, polycystic liver disease, sclerotherapy, bleomycin, treatment outcome, CT-guided interventional radiology

## Abstract

**Objective:**

We aimed to evaluate the long-term efficacy and safety of CT-guided percutaneous aspiration and bleomycin sclerotherapy for symptomatic hepatic cysts at a minimum follow-up of 3 years.

**Materials and methods:**

Medical records of patients who underwent CT-guided percutaneous aspiration and bleomycin sclerotherapy for large (>5 cm) symptomatic simple hepatic cysts and polycystic liver disease were reviewed retrospectively. The choice of percutaneous needle or catheter aspiration was based on the estimated cyst volume. The administered dose of bleomycin was calculated at 5 mg per 100 mL of cyst fluid, with a maximum dose not exceeding 0.6–0.8 mg/kg of body weight per patient. Clinical outcomes, including symptom relief evaluated by the Chronic Liver Disease Questionnaire and the technical response defined by the cyst volume reduction rate, were assessed at 6-, 12-, 24-, and 36-month follow-ups post-sclerotherapy.

**Results:**

A total of 96 cysts in 88 patients (mean age 61.8 ± 18.2 years; 56.8% female) were treated with single-session percutaneous aspiration and bleomycin sclerotherapy. Of these, 81 patients had symptomatic simple hepatic cysts, while 7 had polycystic liver disease. The total bleomycin dose administered ranged from 10 mg to 50 mg per patient, with a median dose of 41.0 mg. All patients reported significant symptom relief, with 81 patients (92.0%) experiencing complete symptom resolution and 7 patients (8.0%) with polycystic liver disease showing improvement from 12 months to 36 months post-procedure. The overall response rates at 6, 12, 24, and 36 months were 96.9% (93 of 96), 100% (96 of 96), 100% (96 of 96), and 100% (96 of 96), respectively. No major complications or bleomycin-related toxicities were observed during or after the procedure.

**Conclusion:**

CT-guided single-session percutaneous aspiration and bleomycin sclerotherapy is an effective and safe treatment for symptomatic simple hepatic cysts and polycystic liver disease.

## Introduction

Cystic liver diseases are commonly encountered in clinical practice as a result of the major advancements and frequently utilization of modern noninvasive imaging techniques. The differential diagnosis for cystic liver diseases is broad and includes simple hepatic cysts, polycystic liver disease, mucinous cystic neoplasms of the liver, Caroli disease, Caroli syndrome, biliary hamartomas and peribiliary cysts, as well as inflammatory or infectious, postraumatic and miscellaneous cysts (1). Macroscopically and microscopically symptomatic simple hepatic cysts and polycystic liver disease share similar characteristics, which are composed of an outer layer of fibrous tissue and are lined by a cuboidal, columnar epithelium that continually produces plasmalike serous fluid, and unconnected to the bile duct system (2). Simple hepatic cysts and polycystic liver disease differ in more than just the number of cysts, and the latter is defined by the presence of >10 hepatic parenchymal cysts that are unconnected to the bile duct system (1).

Image-guided (ultrasound or CT) percutaneous aspiration and sclerotherapy by destroying the epithelium lining the inner surface of the wall to stop intracystic fluid secretion, has been used to treat symptomatic simple hepatic cysts with excellent results regarding the long-term efficacy and safety (3, 4). A variety of sclerosant agents had been used to safely and effectively treat cystic liver lesions, but none of them was considered the best one (3, 4). Bleomycin had emerged as a promising sclerosant agent for the treatment of simple hepatic cysts in the animal experiment and clinical study (5, 6). However, there are limited data on the long-term treatment outcomes of percutaneous bleomycin sclerotherapy for symptomatic simple hepatic cysts and polycystic liver disease in a large number of patients.

The aim of this study was to evaluate the technical aspects and clinical outcomes of CT-guided percutaneous aspiration and bleomycin sclerotherapy for symptomatic hepatic cysts included simple hepatic cysts and polycystic liver disease at a minimum follow-up of 3 years.

## Materials and methods

### Patients

This study was a retrospective interpretation of prospectively collected data and was approved by the institutional review board. Informed consent was obtained from all patients.

Medical records of patients who underwent CT-guided percutaneous aspiration and bleomycin sclerotherapy for symptomatic, simple (non-parasitic and non-neoplastic) liver cysts with clinical, biological and imaging follow-up of at least 3 year between January 2007 and December 2022 were reviewed retrospectively. The inclusion criteria were as follows: (i) Simple hepatic cysts and polycystic liver disease, definitively diagnosed using a combination of imaging techniques (CT, MRI, and ultrasound) according to previously established imaging criteria (1, 7–10); (ii) hepatic cysts larger than 5 centimeters; and (iii) cysts considered symptomatic if the patient experienced any of the following symptoms: non-specific abdominal pain, epigastric mass, discomfort, dyspepsia, dyspnoea or early satiety as well as psychologic distress related to hepatic cysts. Furthermore, the symptoms were assessed by using the chronic liver disease questionnaire (11).

The exclusion criterion was as follows: (i) complex cysts definitively diagnosed using a combination of various imaging techniques (CT, MRI, and ultrasound) according to previously defined imaging criteria (7–10); (ii) patients with severe cardiopulmonary dysfunction, severe hepatic or renal dysfunction, malnutrition, malignant tumors, malignant anemia, unmanageable diabetes, or irremediable coagulation disorders (platelet count <30 × 10^9^/L, prothrombin time ≥30 s, or prothrombin activity <40%); (iii) known allergic response to iodinated contrast medium or the sclerosing agent (ie, bleomycin), or severe allergic diathesis; (iv) lack of a safe percutaneous puncture route; or (v) inability to cooperate or maintain a steady body position during treatment.

### Technique

All aspiration and sclerotherapy procedures were performed with CT guidance (HiSpeed FX/i CT scanner, GE Yokogawa Medical Systems, Tokyo, Japan). To monitor the puncture process, a low-dose protocol of limiting scan coverage for the CT-guided abdominal interventional procedure was used according to the previous literature report (12): Each CT scan was centered around the preconcerted puncture site at 120 kVp, 80 mA per slice, 1.5 pitch, 1-s rotation time, a scan range of 20-mm, and a 10-mm slice thickness with a 5.0-mm reconstruction interval (5 image frames), and a weighted CT dose index of 5.7 mGy. CT fluoroscopy guidance was never used.

On the preoperative diagnostic images, the puncture site and access route were planned based on the location, size, and visibility of the lesions, as well as its relationship with critical anatomical structures. Depending on the previously planned access site and needle trajectory, patients were positioned comfortably in the prone or lateral position with elevated right upper extremity on the CT table. A prone position was never needed. The planning CT scan was performed with laser light guidance and a skin surface grid to identify the skin entry location and define the safest needle approach to reach the lesion. The planned approach should avoid crossing through the gastrointestinal tract or gallbladder, vessels, and pleural cavity. For multiple cysts, either multipoint or single-point multi-angle puncturing planning may need to be performed. Before the procedure, cyst volume was estimated according to the following formula: V = length × width × height × *π*/6.

The skin at the selected access site were sterilized with a standardized antiseptic solution. And then, local anesthesia was achieved using a 25-gage needle and a total of 10 mL of 1% lidocaine: 5 mL of 1% lidocaine was injected into the cutaneous and subcutaneous tissue, and the remaining 5 mL was injected into the liver capsule. Neither conscious sedation nor general anesthesia was administered before the process.

The percutaneous needle or catheter aspiration procedure depended on the estimated cyst volume. Large solitary and multiple cysts with a diameter of 5–10 cm were treated with percutaneous needle aspiration and bleomycin sclerotherapy. Giant cysts larger than 12.5 cm in diameter (i.e., with an estimated cyst volume above 1,000 mL) were treated with percutaneous catheter aspiration and bleomycin sclerotherapy.

**Figure 1:**
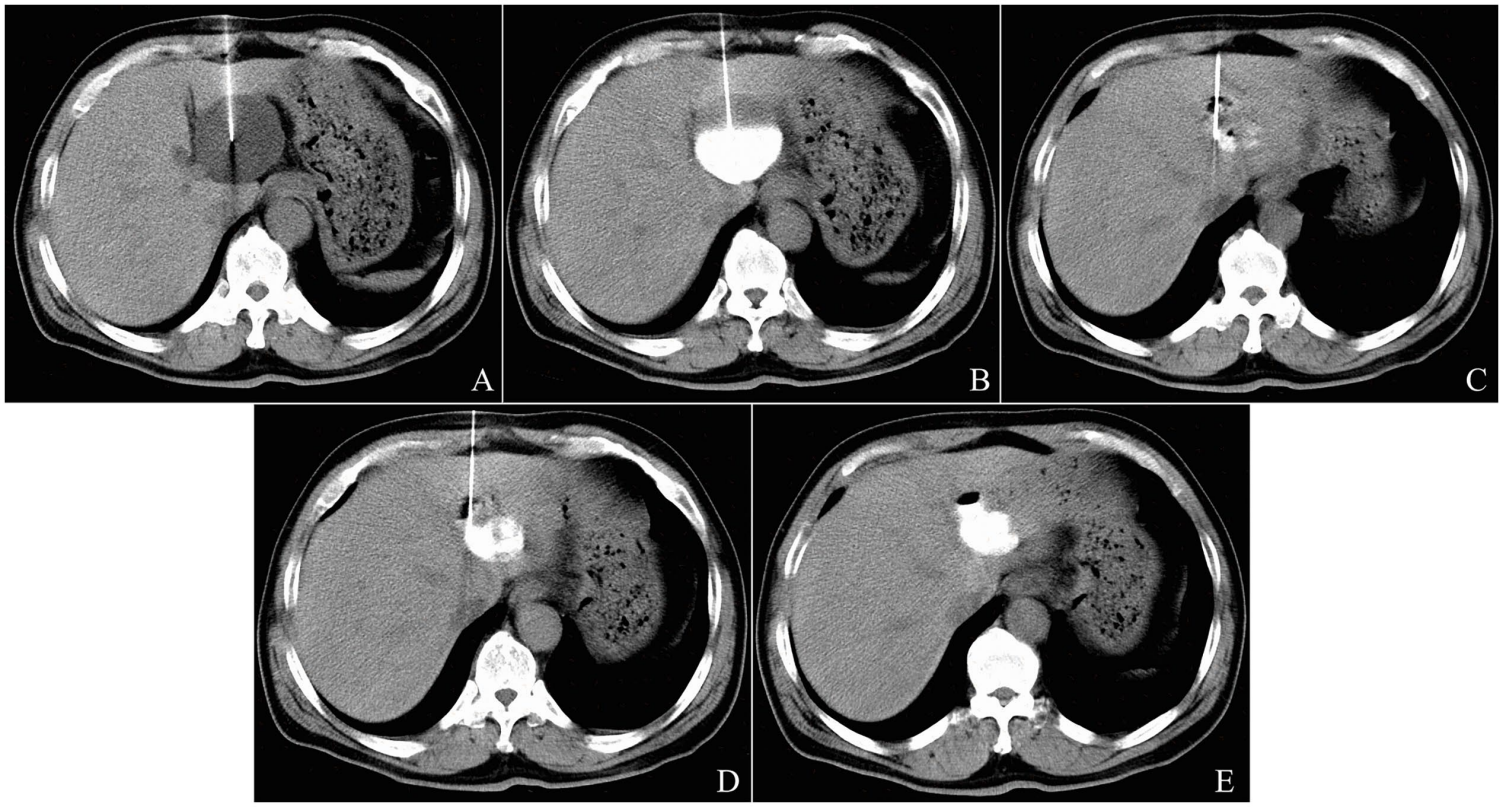
The single-pass single-needle puncturing technique of CT-guided percutaneous needle aspiration and bleomycin sclerotherapy for a 67-year-old male patient with solitary hepatic cyst. A 10-cm 21-gage Chiba needle was inserted into the cyst in the left liver lobe under CT guidance **(A)**. After the first 10 mL of aspirated cyst fluid was sent to the laboratory for examination, 10 mL of diluted contrast media was injected into the cyst to confirm that there was no communication between the cyst and the biliary system **(B)**. The cyst fluid was then evacuated almost completely **(C)**. The repeat CT scan showed no leakage after injection of the half-dose Bleomycin solution contained diluted contrast media **(D)**. The postprocedure CT scan showed that bleomycin solution was stayed in the cyst cavity **(E)**.

Large solitary cysts were punctured directly using a 21-gage Chiba Needle (Cook Medical LLC, Bloomington, United States) with a single-pass along the planned trajectory (Figure 1A). After confirming the correct needle position within the cyst cavity via a controlling CT scan, the needle core is removed, and 10 mL of the cyst content was aspirated for laboratory analysis. Next, 10 mL of undiluted iodinated contrast medium (Iohexol 300 mgI/mL, GE Healthcare, Shanghai, China) was injected into the cyst to ensure no communication between the cyst cavity and the biliary system, and no leakage into the peritoneal cavity (Figure 1B). The remaining cyst fluid with the contrast medium was then aspirated as completely as possible (Figure 1C). The total volume of the aspirated fluid was recorded. Subsequently, bleomycin hydrochloride (Takasaki Plant, Nippon Kayaku Co., Ltd., Tokyo, Japan) was dissolved in diluted contrast medium (1:1 mixture by volume of contrast medium and normal saline solution) at a total dose of 5 mg bleomycin per 100 mL cyst fluid, not to exceed 0.6–0.8 mg/kg body weight per patient. The amount of diluted contrast medium was equivalent to 5–10% of the aspirated fluid volume, not exceeding 10 mL. Bleomycin solution was injected into the aspirated cyst, and remained in the cyst cavity without extraction (Figure 1D). Finally, the needle was removed, and a postprocedure CT scan was performed to rule out leakage from the cyst and possible complications (Figure 1E).

**Figure 2:**
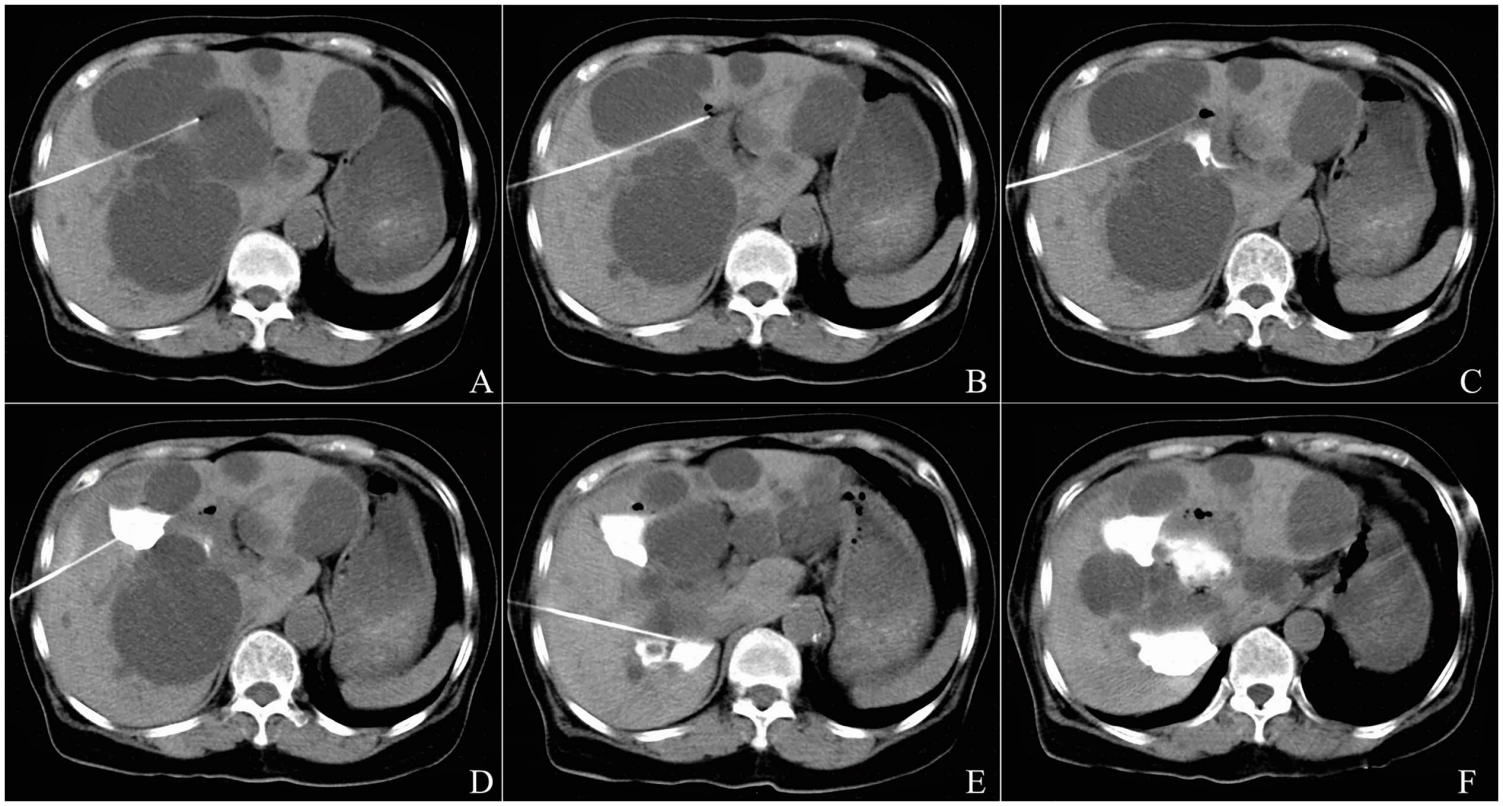
The single-point multi-angulated puncturing technique of CT-guided percutaneous needle aspiration and bleomycin sclerotherapy for three cysts in a 70-year-old female patient with polycystic liver disease. A 15-cm 21-gage Chiba needle was inserted toward the ventral orientation from the right midaxillary line, and went through the cyst located in the lateral section of liver segment V and entered into the cyst located in the medial section of liver segment V **(A)**. The cyst located in the medial section of liver segment V was evacuated completely **(B)**, and bleomycin solution contained diluted contrast media was injected into the cyst cavity without evidence of leakage **(C)**. The needle was then withdrawn into the cyst located in the lateral section of liver segment V, the procedure of aspiration and sclerotherapy was repeated **(D)**. Next, the needle was drawn back to the subcutaneous layer, and than was inserted toward the dorsal orientation from the right midaxillary line and entered into the cyst located in liver segment VI; the procedure of aspiration and sclerotherapy was performed **(E)**. The postprocedure CT scan revealed that bleomycin solution remained behind in three cysts without evidence of spillage **(F)**.

Large multiple cysts could be treated by the single-point, multi-angulated puncturing technique, which involved using the same cutaneous puncture site but adjusting the needle’s insertion angles to successively penetrate each planned cyst (Figure 2). The technological process for treating each cyst was identical to that used for the solitary cyst. The multipoint puncturing technique for multiple cysts was similar to the single-pass, single-needle puncture method, except that the former involved multiple puncture points.

**Figure 3:**
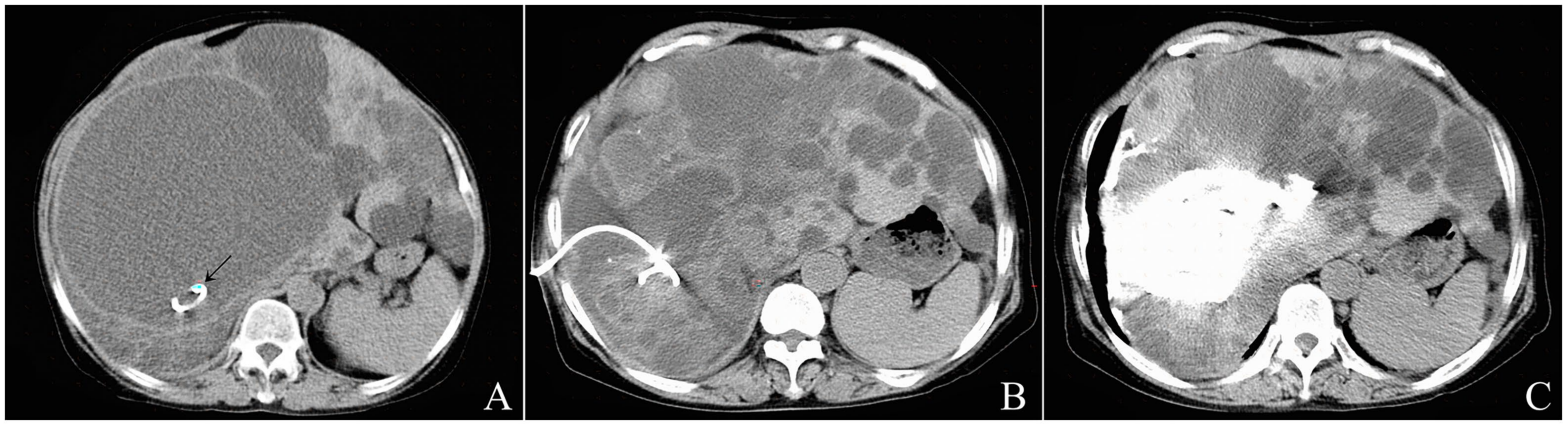
The operation procedure of CT-guided percutaneous catheter aspiration and bleomycin sclerotherapy for a 64-year-old female patient with a giant liver cyst in polycystic liver disease. After inserting percutaneously a 8.5-Fr drainage catheter into the acyst cavity using the Seldinger technique, the position of the tip of central venous catheter (arrowhead) was confirmed by the control CT scan **(A)**. A recheck CT scan on the next day showed complete evacuation of the cyst **(B)**. After injection of bleomycin solution and removal of pigtail catheter, postprocedure CT scan showed nonoccurrence of sclerosant extravasation **(C)**.

Giant cysts were punctured with a 18 G x 7 cm Catheter Access Percutaneous Entry Needle (Cook Medical, Bloomington, IN, United States) using the Seldinger technique. Once the needle tip reached the cyst cavity and was confirmed by CT scan, a 0.035-inch Terumo guidewire (Terumo Corporation, Tokyo, Japan) was threaded freely through the needle without resistance. A 6-Fr Coons tapered dilator (Cook Medical, Bloomington, IN, United States) was then passed over the guidewire to dilate the puncture acess. Subsequently, a 8.5-Fr Multipurpose Drainage Catheter (Cook Medical, Bloomington, IN, United States) was deployed into the cyst cavity over the guidewire. After confirming the position of the pigtail-shaped tip of the drainage catheter within the cyst cavity by a control CT scan (Figure 3A), the cyst fluid was drainaged continuously for 24 h, until no further outflow was observed. A CT scan on the following day confirmed complete evacuation of the cyst (Figure 3B), after which bleomycin sclerotherapy was performed through the drainage catheter as described above (Figure 3C). Following injection of the sclerosant into the cyst cavity, the drainage catheter was withdraw using a guidewire, and the guidewire was then removed.

All procedures involving aspiration and bleomycin sclerotherapy were completed in a single session. The percutaneous needle aspiration procedure were performed on an outpatient basis, while patients undergoing the percutaneous catheter aspiration procedure were required to stay in the hospital for 1 day. After the procedure, patients were continuously monitored with peripheral oxygen saturation, heart rate, respiratory rate and blood pressure for the first 2 h. Patients were positioned in the prone, supine, and left/right lateral decubitus positions for at least 30 min each to ensure sufficient contact of the sclerosant agent with all areas of the cyst wall.

### Follow-up

Patients were followed up at 6, and 12 months after sclerotherapy, and then annually. During each visit, clinical assessments were conducted, including the presence of subjective symptoms, liver function tests, and measurement of the size of the treated hepatic cyst.

Symptoms was assessed using the Chronic Liver Disease Questionnaire (11), and categorized as “disappearance,” “improvement’, “no change,” or “aggravation” of symptoms (13). Treatment was considered effective if patients were classified as “disappearance” and “improvement.” The interval time between the procedure and symptom relief was recorded during follow-up visits.

The cyst volume was estimated according to the orthogonal diameter of the cyst obtained from ultrasound, CT or MRI examinations. The volume reduction rate (VRR) is calculated as follows: VRR = (initial volume − final volume)/initial volume × 100% after treatment. The treatment responses were classified into four categories as follows: complete regression (CR; ie, invisible or immeasurable), near-CR (ie, VRR > 85%), partial regression (PR; ie, VRR of 50–85%), and no response (NR; ie, VRR < 50%). The overall response rate was defined as the percentage of patients in whom CR, near-CR, or PR were achieved (11).

### Data collection

Data collected included patient demographics, clinical assessments, imaging results, treatment processes, treatments complications, and therapeutic outcomes. Complications were classified as minor or major according to the definitions established by the Society of Interventional Radiology (14).

### Statistical analysis

Baseline characteristics are presented as mean (±SD) or median (±IQR), depending on the distribution of the data. All statistical analyses were performed using the Statistical Package for Social Sciences (SPSS), version 25 (IBM Corp, Armonk, NY, United States). *p*-values were two-tailed and a value < 0.05 was considered statistically significant.

## Results

### Clinical characteristics of patients

This study included 96 symptomatic hepatic cysts in 88 patients who underwent CT-guided single-session percutaneous aspiration and bleomycin sclerotherapy from January 2007 to December 2022, with follow-up lasting more than 3 year. Demographic, clinical, and imaging characteristics of patients with symptomatic hepatic cysts were shown in Table 1.

**Table 1:** Demographic, clinical, and imaging characteristics of 96 symptomatic hepatic cysts in 88 patients.

Characteristics	Value
Number of patients (n)	88
Age (y)	
Mean ± SD	61.8 ± 18.2
Range	35–86
Sex	
Male	38 (43.2%)
Female	50 (56.8%)
Symptoms*	
Abdominal symptoms	
Abdominal bloating	19 (21.6%)
Abdominal pain	35 (39.7%)
Abdominal discomfort	33 (37.5%)
Systemic symptoms	0
Fatigue	2 (2.3%)
Emotional dysfunction	11 (12.5%)
Worry	52 (59.1%)
Cyst number	
Solitary cyst	73 (82.9%)
Multiple cysts	8 (9.1%)
Polycystic liver disease	7 (7.9%)
Hepatic cysts treated (n)	96
Cyst location (n)	
Right lobe (n)	63 (65.6%)
Left lobe (n)	33 (34.4%)
Cyst size (cm)	
Mean ± SD	12.0 ± 14.8
Range	5.0–24.0

*According to the chronic liver disease questionnaire, symptoms were classified into six scales including abdominal symptoms, fatigue, systemic symptoms, activity, emotional function and worry (11).

The patients included 50 women (56.8%) and 38 men (43.2%) with a mean age of 61.8 ± 18.2 years (age range, 35–86 years). Seventy-three patients (79%) had solitary cysts, and eight patients (9.1%) had multiple cysts, and seven patients (7.9%) had polycystic liver disease, which was defined as the presence of more than 10 hepatic cysts scattered throughout the liver (1). The cysts were located in the right lobe in 63 patients (65.6%) and in the left lobe in 33 patients (34.4%). The mean diameter of the hepatic cysts was 12.0 ± 14.8 cm (range, 5.0–24.0 cm). All patients presented with abdominal symptoms at diagnosis, including abdominal bloating in 19 cases (21.6%), and abdominal pain in 35 cases (39.7%) and abdominal discomfort in 33 cases (37.5%). Additionally, some patients reported psychological symptoms, including fatigue in 2 cases (2.3%), and emotional dysfunction in 11 cases (12.5%) and worry in 52 cases (59.1%),

### Technical aspects of bleomycin sclerotherapy

Sclerotherapy was performed in a single session using the single-pass single-needle puncturing technique in 73 patients (83.0%), and the single-point multi-angle puncturing technique in five patients (5.7%), and the multi-point puncturing technique in three patients (3.4%), and the percutaneous catheter aspiration technique in seven patients (8.0%). Neither the biliary duct nor the liver vessel were visualized during intracystic contrast medium injection. The mean volume of hepatic cysts measured by abdominal ultrasound or CT images obtained before percutaneous sclerotherapy was 980.0 mL (range, 62.5–4257.0 mL), while the mean volume of the aspirate was 860 mL (range, 60.0–4100.0 mL). The ratio of aspirate volume to calculated cyst volume was 87.8% (range, 74.7–96.3%). Aspirated fluids from all cysts were classified as transudate, with no malignant cells on cytologic examination. Biochemical analysis showed normal levels of lactic dehydrogenase, protein, amylase, glucose, electrolytes, urea nitrogen, and creatinine levels. The total dose of bleomycin administered ranged from 10 mg to 50 mg per patient, with a median of 41.0 mg per patient, dissolved in diluted contrast medium ranging in amount from 1 mL to 10 mL, with an average of 8.5 mL per patient.

### Clinical outcomes

The details of follow-up and outcome were summarized in Table 2. At 6-month follow-up, symptoms disappearance was observed in 53 patients (60.2%), while improvement was noted in 25 patients (28.4%), and no change was reported in 10 patients (11.4%). The self-reported symptom information remained consistent from 12 months to 36 months after the procedure: symptom disappearance occurred in 81 patients (92.0%), and improvement was noted in seven patients (8.0%), the latter being patients with polycystic liver disease. Cyst volume at each follow-up timepoint decreased significantly compared to pre-treatment measurements (all *p* < 0.001). A significant reduction was also observed between the 6-month and the 12-month follow-up (*p* < 0.01). However, no significant difference in cyst volume were observed among the 12-, 24-, and 36-month follow-up assessments (e.g., 12 vs. 36 months, *p* = 0.75), indicating that the maximum therapeutic effect was achieved and stabilized by 12 months post-procedure.

**Table 2:** Three-year treatment outcomes of percutaneous aspiration and bleomycin sclerotherapy for symptomatic hepatic cysts.

Treatment outcomes	Follow-up times (months)				
	Before treatment	6	12	24	36
Symptoms (*n* = 88)					
Disappearance (%)		53 (60.2)	81 (92.0)	81 (92.0)	81 (92.0)
Improvement (%)		25 (28.4)	7 (8.0)	7 (8.0)	7 (8.0)
No change (%)		10 (11.4)	0 (0.0)	0 (0.0)	0 (0.0)
Aggravation (%)		0 (0.0)	0 (0.0)	0 (0.0)	0 (0.0)
Cyst volume (mL) (*n* = 96)					
Mean ± SD	980.0 ± 735.4	*379.6* ± 157.7*	14.7 ± 10.4*^†^	13.5 ± 11.3*^†§^	13.0 ± 17.5*^†§^
Range	62.5–4257.0	0.0–610.0	0.0–79.2	0.0–75.6	0.0–75.6
Treatment responses* (*n* = 96)					
CR (%)		11 (11.4)	14 (14.6)	14 (14.6)	14 (14.6)
near-CR (%)		20 (20.8)	67 (69.8)	68 (70.8)	68 (70.8)
PR (%)		62 (64.6)	15 (15.6)	14 (14.6)	14 (14.6)
NR (%)		3 (3.1)	0 (0.0)	0 (0.0)	0 (0.0)

^*^P < 0.001 for all post-treatment volumes compared to pre-treatment volume.

^†^*P* < 0.01 compared to the cyst volume at 6 months after treatment.

^§^Not significant (*P* > 0.05) compared to the cyst volume at 12 months after treatment.

CR, complete regression; near-CR, near-complete regression; PR, partial regression; NR, no response.

**Figure 4:**
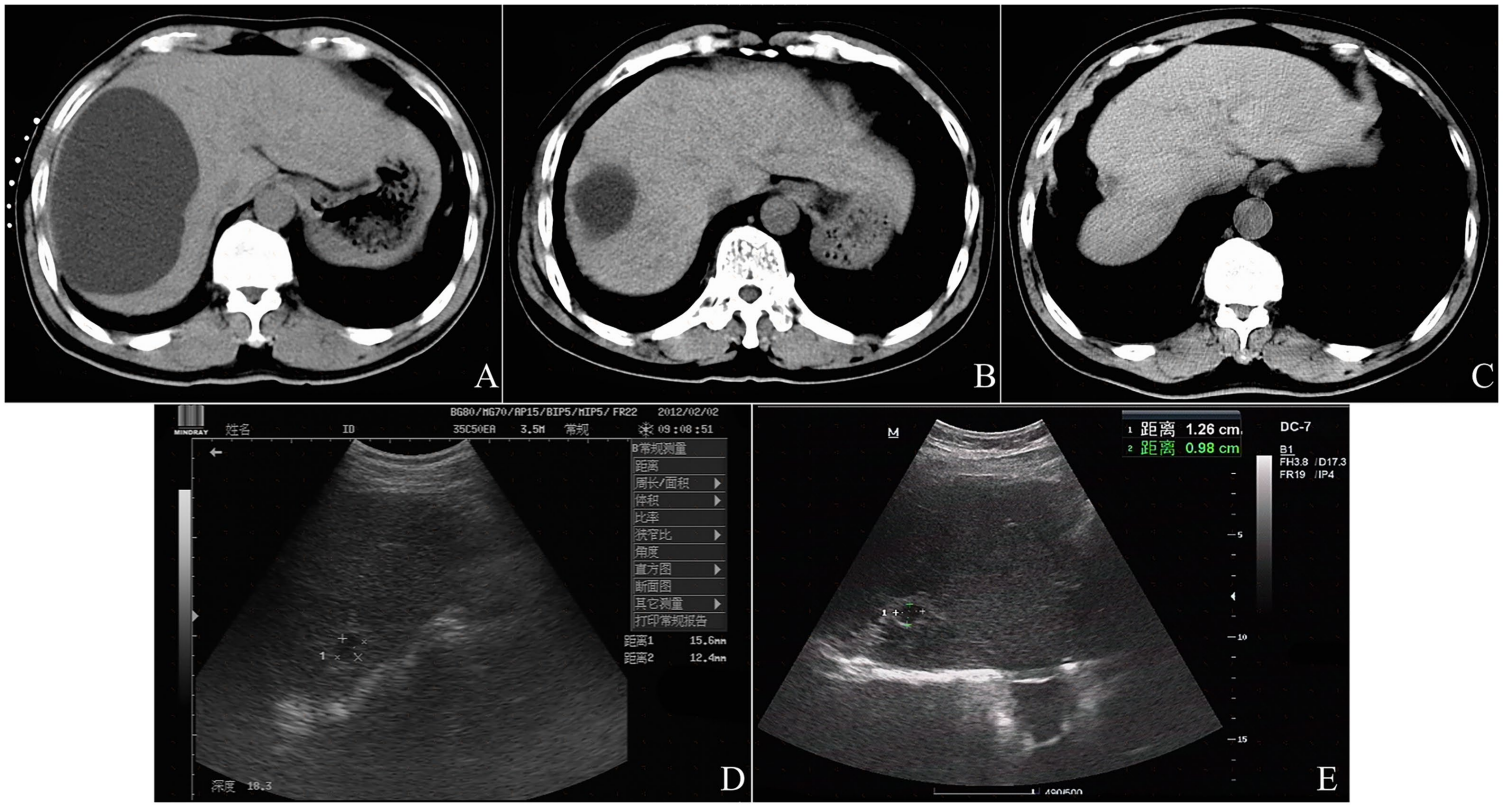
A typical example of the imaging-based treatment response after CT-guided percutaneous aspiration and bleomycin sclerotherapy in a 69-year-old man with a simple left renal cyst. Preprocedural CT scan shows an initial cyst volume of 554.4 mL (12.6 cm × 8.8 cm × 10.0 cm in size) **(A)**. At 6-month follow-up, CT shows a cyst volume of 34.3 mL (4.9 cm × 3.5 cm × 4.0 cm in size) and the volume reduction rate (VRR) of 93.8% **(B)**. At 12-month follow-up, CT shows a cyst volume of 1.7 mL (20.0 mm × 13.3 mm × 13.0 mm in size) and the volume reduction rate (VRR) of 99.7% **(C)**. At 24-month follow-up, US shows a cyst volume of 1.2 mL (15.6 mm × 12.4 mm × 13.0 mm in size) and the volume reduction rate (VRR) of 99.8% **(D)**. At 36-month follow-up, US shows a cyst volume of 0.7 mL (12.6 mm × 9.8 mm × 12.0 mm in size) and the volume reduction rate (VRR) of 99.9% **(E)**.

Regarding imaging-based treatment responses, the cysts refilled partially in the initial stage after sclerotherapy and decreased gradually in size throughout the entire follow-up period, without significantly changes since 12-months after the procedure (Figure 4). At the 6-month follow-up, CR was observed in 11 cysts (11.4%), and near-CR in 20 cysts (20.8%), and PR (%) in 62 cysts (64.6%), and NR in 3 cysts (3.1%). At 12-month follow-up, 14 cysts (14.6%) achieved CR, 67 cysts (69.8%) achieved near-CR, and 15 cysts (15.6%) achieved PR. At 24- and 36-month follow-up, 14 cysts (14.6%) achieved CR, 68 cysts (70.8%) achieved near-CR, and 14 cysts (14.6%) achieved PR.

### Adverse events

No patients reported significant pain during the procedure. There were no major complications or toxicities associated with bleomycin occurred during or after the procedure. Minor complications included low-grade fever ranging from 37.3 to 39 °C for 3 days in 11 patients (12.5%), which resolved without treatment. Liver function tests showed no abnormality.

## Discussion

To the best of our knowledge, the present study is the first to evaluate the technical aspects and long-term clinical outcomes of CT-guided percutaneous aspiration and bleomycin sclerotherapy for symptomatic hepatic cysts. The findings indicate that the long-term efficacy of a single session of bleomycin sclerotherapy was excellent, as cyst volume decreased significantly and all patients reported either symptom disappearance or improvement with a minimum follow-up of 3 years. However, the findings of this study should be interpreted in the context of its limitations. Firstly, the retrospective, single-arm design, while suitable for evaluating the long-term outcomes of a specific technique, inherently lacks a control group for direct comparison. Therefore, we cannot make definitive claims regarding the superiority of bleomycin over other established sclerosants, such as ethanol, or of CT-guidance over alternative imaging modalities like ultrasound. The excellent outcomes observed herein warrant future investigation through prospective, randomized controlled trials to conduct such head-to-head comparisons.

Technical essentials of the CT-guided percutaneous aspiration and sclerotherapy for hepatic cysts have been previously described in the literatures (15, 16). Notably, three key technical issues should be highlighted about this study. First, a low-dose protocol of limiting scan coverage for the CT-guided abdominal interventional procedure was performed to decrease the risk of radiation. According to the definitions of the volume CT dose index and the dose-length product, strategies such as limiting scan coverage to only anatomical structures of clinical interest and minimizing the number of acquisition phases for multi-phase examination are effective for dose reduction (17, 18). Second, the single-point multi-angulated puncturing technique for large multiple cysts was employed to decrease puncture numbers and enhance patient comfort. Proper planing the access route based on pre-procedural radiological examinations and skilled operation techniques is crucial for the success rate of percutaneous puncture procedures. It is important to noted that the shape and location of the latter cyst may change after aspiration of a prior cyst. Third, percutaneous catheter aspiration for giant cysts larger than 12.5 mm in diameter (namely, the estimated cyst volume were above 1,000 mL) was aimed at reducing procedure duration, improving patient compliance, and preventing the sudden decrease in abdominal pressure.

The sclerosing mechanisms of intracyst bleomycin injection may be resemble those of pulmonary fibrosis induced by intratracheal bleomycin instillation. Numerous experimental studies and clinical findings indicate that bleomycin can cause pulmonary vascular endothelial cell damage independently of its DNA effects through lipid peroxidation, leading to immune cell infiltration and fibroblast proliferation (19–22). A recent experimental study demonstrated that histopathological changes after bleomycin sclerotherapy in rabbit gallbladders, used as a model for simple hepatic cysts, were characterized by sequential epithelial destruction, inflammatory cell infiltration, collagen proliferation and epithelial partial regeneration (6). Epithelial degeneration was observed as early as day one, with mucosa disappeared at 7 days and mucosal structures absent from days 14–84 after intracyst bleomycin injection. Inflammatory infiltration peaked at the first day after bleomycin sclerotherapy and persisted throughout the experiment, while collagenous proliferation was evident by day 7, gradually decreasing thereafter (6). Although further investigation into the cellular and molecular mechanisms of intracystic bleomycin injection is needed, these results from animal studies can help explain the clinical outcomes of bleomycin sclerotherapy.

The safety of intralesional bleomycin sclerotherapy primarily depends on the administered dose, because pulmonary toxicity as the most serious complications of bleomycin remains a dose-limiting toxicity (22). Although an optimal dose for percutaneous bleomycin sclerotherapy has not yet been universally agreed upon, it is generally accepted that the cumulative lifetime dose of bleomycin should not exceed 400 mg or 5 mg/kg to limit the risk of pulmonary fibrosis (23–27). The recommended dose for intralesional bleomycin injection in vascular anomalies is 0.5–1 mg/kg/dose or summated dose should not exceed 5 mg/kg, with varied according to the size of the lesion (27–30). Pharmacokinetic analysis of bleomycin sclerotherapy in patients with vascular malformations revealed terminal elimination half-life of 92.84 (±34.39) minutes, and the volume of distribution of 3.44 (±5.06) L, and total systemic exposure area under the curve of 78.99 (±62.84) mg min/L (31). This study indicates that bleomycin is systemically absorbed when used as a sclerosant, whether injected either in the lumen or interstitial space of the involved lesion (31). However, evidence suggests that routine laboratory assessments of immune and liver function following intralesional bleomycin sclerotherapy may not be necessary, as clinically relevant abnormalities are unlikely to be detected (32). While the pharmacokinetics of bleomycin following intralesional bleomycin injection for cystic lesions has never been reported, systemic absorption is expected to be lower compared to vascular malformations due to differences in blood supply. Systemic toxicity reports following intralesional bleomycin injection for cystic lesions are limited (5, 6, 12, 23, 25, 28, 30). In the present study, the administered dose of bleomycin was calculated at 5 mg bleomycin per 100 mL of cyst fluid, not to exceed 0.6–0.8 mg/kg body weight per patient. The total dose of bleomycin administered ranged from 10 mg to 50 mg per patient, with a median of 41.0 mg per patient. No major complications or toxicities associated with bleomycin were observed during or after the procedure. It is important to note that the use of bleomycin for the sclerotherapy of hepatic cysts is an off-label application. Nonetheless, a growing body of clinical evidence, including the present study, supports its efficacy and favorable safety profile when administered at the recommended dosages, which are well below the cumulative lifetime dose associated with pulmonary toxicity (23–27). This usage is increasingly recognized within the field of interventional radiology for the management of various cystic and vascular anomalies.

The significantly satisfactory treatment outcomes from intralesional bleomycin sclerotherapy have been documented in the published literature (5, 6, 12, 24–27, 29–31). In the present study, the self-reported symptoms disappeared in 53 patients (60.2%), and improved in 25 patients (28.4%), and no change in 10 patients (11.4%) at 6-month follow-up; symptoms disappeared in 81 patients (92.0%) and improved in seven patients (8.0%) from 12 months to 36 months follow-up after the procedure, with the latter just be the patients with polycystic liver disease. Cyst volume at each follow-up timepoint decreased significantly compared to pre-treatment measurements (*p* < 0.01), with the cyst size refilling partially from 980.0 ± 735.4 mL before the procedure to 379.6 ± 157.7 mL at 6-month follow-up (*p* < 0.01), and decreasing gradually to 14.7 ± 10.4 mL at 12-month, and 13.5 ± 11.3 mL at 24-month and 13.0 ± 17.5 mL at 36-monthp, with no significant differences among follow-up timepoints from 12 months onward (*p* > 0.05). At 6-month follow-up, the overall response rate was 96.9% (93 of 96), with CR in 11 cysts (11.4%), near-CR in 20 cysts (20.8%), PR in 62 cysts (64.6%), and NR in 3 cysts (3.1%). At 12-month follow-up, the overall response rate was 100% (96 of 96), with CR in 14 cysts (14.6%), near-CR in 67 cysts (69.8%), and PR in 15 cysts (15.6%). Fifteen cysts (15.6%). At 24- and 36-month follow-up, the overall response rate was 100% (96 of 96), with CR in 14 cysts (14.6%), near-CR in 68 cysts (70.8%), and PR in 14 cysts (14.6%). The results suggest that the sclerosing effect of intracyst bleomycin injection evolves gradually, consistent with findings from the previous experimental study (6) and clinical report (12). They also indicate that the optimal follow-up time for assessing the clinical outcomes should be scheduled at 12 months after intracyst bleomycin injection, as longer-term follow-up may not be necessary in terms of cost-effectiveness.

The present study has several limitations. Firstly, its retrospective observational design and the lack of a control group for comparison with other sclerosants or guidance techniques may introduce selection bias and limit the generalizability of the findings. Secondly, the use of bleomycin for sclerotherapy is technically off-label, although its clinical utility is supported by numerous studies. Thirdly, while we have incorporated additional statistical comparisons of cyst volume over time, the absence of a comparative control group precludes more robust statistical analyses. Finally, attention should be paid to radiation exposure during CT-guided interventions, necessitating appropriate adjustments to CT scanner parameters to minimize radiation doses without significantly prolonging procedure time or degrading image quality.

In conclusion, CT-guided single-session percutaneous aspiration and single-injection bleomycin sclerotherapy for symptomatic simple hepatic cysts and polycystic liver disease is a potent and safe therapeutic alternative that demonstrates excellent long-term efficacy without significant complications. Multicenter prospective randomized controlled studies with longer term follow-up and larger sample sizes are needed to further establish the clinical evidence for the clinical benefits of intralesional bleomycin sclerotherapy for symptomatic hepatic cysts.

## Data Availability

The raw data supporting the conclusions of this article will be made available by the authors, without undue reservation.
